# Tumor immune cell clustering and its association with survival in African American women with ovarian cancer

**DOI:** 10.1371/journal.pcbi.1009900

**Published:** 2022-03-02

**Authors:** Christopher Wilson, Alex C. Soupir, Ram Thapa, Jordan Creed, Jonathan Nguyen, Carlos Moran Segura, Travis Gerke, Joellen M. Schildkraut, Lauren C. Peres, Brooke L. Fridley

**Affiliations:** 1 Department of Biostatistics and Bioinformatics, Moffitt Cancer Center, Tampa, Florida, United States of America; 2 Department of Health Informatics, Moffitt Cancer Center, Tampa, Florida, United States of America; 3 Department of Pathology, Moffitt Cancer Center, Tampa, Florida, United States of America; 4 Department of Epidemiology, Emory, Atlanta, Georgia, United States of America; 5 Department of Cancer Epidemiology, Moffitt Cancer Center, Tampa, Florida, United States of America; Johns Hopkins University - Homewood Campus: Johns Hopkins University, UNITED STATES

## Abstract

New technologies, such as multiplex immunofluorescence microscopy (mIF), are being developed and used for the assessment and visualization of the tumor immune microenvironment (TIME). These assays produce not only an estimate of the abundance of immune cells in the TIME, but also their spatial locations. However, there are currently few approaches to analyze the spatial context of the TIME. Therefore, we have developed a framework for the spatial analysis of the TIME using Ripley’s *K*, coupled with a permutation-based framework to estimate and measure the departure from complete spatial randomness (CSR) as a measure of the interactions between immune cells. This approach was then applied to epithelial ovarian cancer (EOC) using mIF collected on intra-tumoral regions of interest (ROIs) and tissue microarrays (TMAs) from 160 high-grade serous ovarian carcinoma patients in the African American Cancer Epidemiology Study (AACES) (94 subjects on TMAs resulting in 263 tissue cores; 93 subjects with 260 ROIs; 27 subjects with both TMA and ROI data). Cox proportional hazard models were constructed to determine the association of abundance and spatial clustering of tumor-infiltrating lymphocytes (CD3+), cytotoxic T-cells (CD8+CD3+), and regulatory T-cells (CD3+FoxP3+) with overall survival. Analysis was done on TMA and ROIs, treating the TMA data as validation of the findings from the ROIs. We found that EOC patients with high abundance and low spatial clustering of tumor-infiltrating lymphocytes and T-cell subsets in their tumors had the best overall survival. Additionally, patients with EOC tumors displaying high co-occurrence of cytotoxic T-cells and regulatory T-cells had the best overall survival. Grouping women with ovarian cancer based on both cell abundance and spatial contexture showed better discrimination for survival than grouping ovarian cancer cases only by cell abundance. These findings underscore the prognostic importance of evaluating not only immune cell abundance but also the spatial contexture of the immune cells in the TIME. In conclusion, the application of this spatial analysis framework to the study of the TIME could lead to the identification of immune content and spatial architecture that could aid in the determination of patients that are likely to respond to immunotherapies.

## Author summary

New technologies, such as multiplex immunofluorescence microscopy, are being developed and used for the assessment and visualization of the tumor immune microenvironment (TIME). These assays produce not only an estimate of the abundance of immune cells in the TIME, but also their spatial locations; however, there are currently few approaches to analyze the spatial context of the TIME. Thus, we have developed a framework for the spatial analysis of the TIME and applied this method to the analysis of T-cells collected from patients with high-grade serous ovarian carcinoma in the African American Cancer Epidemiology Study. We found that patients with high abundance and low spatial clustering of tumor-infiltrating lymphocytes and T-cell subsets in their tumors had the best overall survival. Additionally, best survival was observed for patients with tumors displaying high co-occurrence of cytotoxic T-cells and regulatory T-cells. These findings underscore the prognostic importance of evaluating not only immune cell abundance but also the spatial contexture of the immune cells in the ovarian TIME. The use of our framework for spatial analysis of the TIME and immune cell clustering may be applicable in other cancers and provide a novel approach to identification of biomarkers for predicting patient outcomes.

This is a *PLOS Computational Biology* Methods paper.

## 1. Introduction

Immunology has been a break-through area in the treatment of cancer [1,2]. One of the most important findings is the use of agents to block immune checkpoints to activate anti-tumor immunity. Immune checkpoints are the mechanism by which the immune system maintains self-tolerance. That is, immune checkpoints are regulators of the immune system and prevent the immune system from attacking “good” cells. In the case of cancer, the cancerous cells hijack this mechanism to protect themselves from being attacked by the immune system [3]. The use of checkpoint inhibitors in the treatment of cancer has been a revolutionary approach and has resulted in the development of numerous checkpoint inhibitors, such as CTLA-4 and PD-1/PD-L1 inhibitors [4].

Tumors with a dense infiltration of lymphocytes, also known as tumor-infiltrating lymphocytes (TILs), are consistently associated with more favorable outcomes among cancer patients [5–7]. However, abundance alone may not explain a patient’s clinical outcome, and consideration of the spatial architecture of the tumor immune microenvironment (TIME) may shed new light on clinical outcomes and response to immunotherapies. Lee, et al. (2020) showed that diffuse large B cell lymphoma tumors with similar densities of TILs had heterogeneous spatial patterns of cytotoxic T-cells [8], and a study in colorectal cancer observed that cell-to-cell distances and spatial heterogeneity were more promising as prognostic biomarkers than cell densities [9]. More recently, as study by Steinhart et al (2021) found that co-localization of tumor-associated macrophages and B cells or CD4 T cells was significant associated with better survival from ovarian cancer [10].

**Fig 1:**
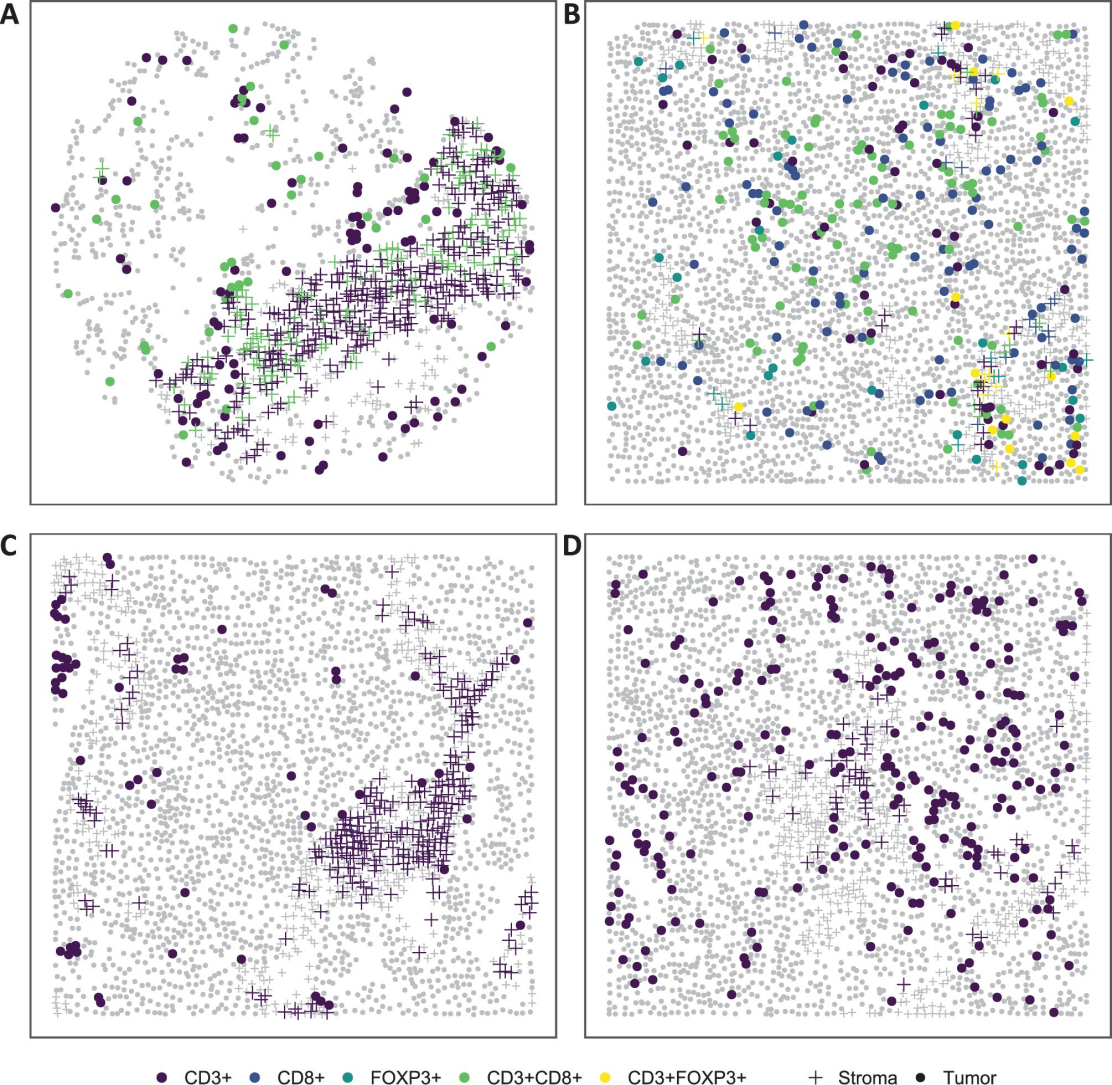
Example of IF data from (A) a TMA core sample and (B) an intra-tumoral region of interest (ROI). As illustrated in the two example figures, TMAs tend to have more “holes” and uneven cell density as compared to ROIs. Examples of clustering patterns observed in the intra-tumoral ROIs; (C) locations of CD3+ cells in which the pattern deviates from complete spatial randomness (CSR); and (D) location of CD3+ cells in which the pattern is close to CSR.

Many technologies have been developed to study the TIME. One study approach is the use of multiplex immunofluorescence (mIF) microscopy which provides both a summary of the number of cells positive for a given immune marker (e.g., abundance or density) and spatial locations of cells. By having spatial locations of the cells positive for the various immune markers, one can determine spatial clustering and co-occurrence of immune cells. mIF can be applied to both regions of interest (ROIs) selected from a stained tumor slide or tissue microarrays (TMAs) [11]. Many challenges arise with the use of data resulting from TMAs. In particular, the tissue area can become folded or ripped due to the “slicing” done for different experiments, leading to imperfections in the shape and the ability to measure all cells in the area. These imperfections can lead to TMAs that have sections that appear like no cells exist, as depicted in **Fig 1A**. In contrast, ROIs typically do not exhibit this artifact in the data acquisition process (**Fig 1B**).

The most common analysis of data from the TIME involves the use of the summary measures representing immune content in the entire sample (i.e., proportion of CD3+ cells, density). However, this type of analysis ignores the spatial architecture of the immune cells within the tumor, which can vary between tumors. As illustrated in **Fig 1**, some tumors show clustering of TILs (CD3+ cells; **Fig 1C**), while other tumors show more dispersion of TILs **(Fig 1D)**. While there have been studies that attempt to describe the relationship between spatial clustering of immune cells and patient outcomes using such measures as nearest-neighbor distance (NND) [12], Hypothesized Interaction Distribution (HID) [13,14], Morisita-Horn index [15], and Mander’s correlation coefficient [16], many approaches fail to account for issues related to: correlation between spatial and abundance measures of immune cells; edge/border effects; and regions in which no cells were able to be measured [17,18]. Thus, we have developed a framework for the spatial analysis of the TIME using Ripley’s *K* [19], coupled with a permutation-based framework to estimate and measure the departure from complete spatial randomness (CSR) as a measure of the relationships and interactions between immune cells. We applied this analysis framework to epithelial ovarian cancer (EOC), the deadliest gynecologic malignancy in the U.S. [20]. Specifically, we characterized the TIME and explored links between immune cell abundance of T-cells and their spatial characteristics with overall survival (OS) among EOC patients.

## 2. Methods and materials

### 2.1. Ethics statement

All participants provided verbal consent at the time of the baseline telephone interview, and written informed consent was obtained for the procurement of tissue specimens and collection of medical records. The parent protocol for the AACES study was approved by the Duke Health Institutional Review Board (IRB number Pro00022451).

### 2.2. Study population and immunofluorescence assays

To test out the proposed framework for assessing spatial clustering within the TIME using Ripley’s *K*, we used the data from the African American Cancer Epidemiology Study (AACES). AACES is a population-based case-control study of 595 African American (AA) women with EOC residing in 11 geographic locations in the U.S. and 752 controls enrolled between December 2010 and August 2016 [21]. Cases were identified through cancer registries and hospitals, and were eligible for the study if they were 1) aged 20–79 years, 2) self-reported AA race, and 3) resided in one of the 11 geographic locations. Study participants completed a telephone survey at baseline, and for ~90% of the cases, formalin-fixed paraffin-embedded (FFPE) tissue blocks of the primary tumor were procured. For 75% of cases with tissue, twenty-five sections were cut from each tissue block, and TMAs were created for the other 25%. A centralized pathology review was completed to confirm diagnosis and histology. A systematic collection of vital status and follow-up data through linkages with cancer registries and the National Death Index (NDI) has been conducted annually. As the distribution of immune cells in the tumor microenvironment and their association with outcomes differ according to histotype [22], we focused on the most common and one of the deadliest histotypes, high-grade serous carcinoma (HGSC) [23], to limit contributions of disease heterogeneity.

To determine immune profiles of these tumors, mIF staining was completed using the Opal chemistry and multispectral microscopy Vectra system (Akoya Biosciences). Tumors were stained for one panel including seven fluorophore-labeled markers: CD3, CD8, FOXP3, CD11b, CD15, DAPI and pancytokeratin (PanCK). After staining, slides were scanned, and image capture was performed with the Vectra 3.0 Automated Quantitative Pathology Imaging System (Akoya Biosciences) with images are exported from InForm (Akoya Biosciences) and loaded into HALO (Indica Labs, New Mexico) for quantitative image analysis. Coordinates of the cell locations are based on pixels of the image where the image resolution is 0.4977 microns per pixel (Mpp). For the statistical analyses, we focused on tumor-infiltrating lymphocytes (CD3+) and relevant T-cell subsets (CD3+CD8+ cytotoxic T-cells; CD3+FOXP3+ regulatory T-cells). In addition to TMAs, mIF was performed on whole slide images, where three ROIs from the intratumoral region of each tissue section were selected for analysis (e.g., 100% tumor cells by cellularity and PCK expression). A summary of the study participants is presented in **S1 Table**, where 94 subjects were on TMAs (263 samples) and 93 subjects had ROIs (260 samples), with 27 subjects with both TMA and ROI data. From the 27 patients that we have both ROIs and TMAs, there are 72 intratumoral ROIs, and 75 TMA cores.

### 2.3. Ripley’s *K* and complete spatial randomness

In our proposed framework, the locations of the immune positive cells in the TIME can be thought of as a spatial point process. The arrangement of these cells may not follow the assumption of complete spatial randomness (CSR) for homogenous spatial processes, where positive cells occur at the same rate *λ* for the entire region. Attraction (clustering), repulsion, and competition (dispersion) are all examples of interactions that would lead to a violation of CSR. Ripley’s *K* (19) is a popular measures to quantify these interactions by counting the number of neighboring cells within a radius for each positive immune cell, normalizing by the maximum number of pairwise distances, and correcting for border effects. **Fig 1D** shows an ROI that exhibits a spatial process that is close to CSR, while **Fig 1C** shows an ROI that violates the CSR assumption.

Ripley’s *K* is computed at several rings with varying radii, *r*, that is given by the following formula:

$$\hat{K}\;(r)=(n{\left(n-1)\right)}^{-1}A\sum_{i=1}^{n}\sum_{j\neq i}w_{ij}1(|\left(x_{j}:d(x_{i},x_{j})<r\right)|),$$

where *n* is the number of cells, *A* represents the area of the region of interest within the TMA or ROI, *w*_*ij*_ corresponds to the edge correction, and **1**|*x*_*j*_:*d*(*x*_*i*_,*x*_*j*_)<*r*| is an indicates whether the *j*^*th*^ is a neighbor of the *i*^*th*^ cell (the Euclidean distance between cells *i* and *j* is less than *r*), where the expected value for $\hat{K}(r)=\pi r^{2}$. The edge correction accounts for undercounting of the number of neighboring cells when a cell on the periphery of the TMA core or ROI. The difference between the observed and expected value, $E(\hat{K}(r))=\lambda^{-1}E(n)=\pi r^{2}$ where *E*(*n*) is the expected number of cells and *λ* is the intensity of the cell, helps determine the degree of regularity or clustering. A positive difference corresponds to a higher degree of clustering than expected, while a negative difference is evidence of a regular pattern existing [19,24,25]. Ripley’s *K* can be calculated in a univariate form as described above, or a bivariate form where co-occurrence of immune cell types is explored, for example, the spatial co-localization of cytotoxic T-cells and regulatory T-cells.

Ideally, these point processes occur in a rectangular or circular region of space, however, the region where cells appear are not necessarily perfect rectangles or circles, such as in the case of TMAs. To that end, we can still estimate Ripley’s *K* over the convex hull of the cells measured in the sample. Another challenge is the “edge effect”, where cells on the periphery of the tissue sample lose neighboring cells that are located outside the sampled region. Two common edge corrections are called isotropic and translation [26,27]. The translation and isotropic edge correction methods can accommodate settings when there are a small number of positive cells in the tissue samples [24]. Additionally, little difference was observed in the estimate of Ripley’s *K* using these two border correction methods to mIF data, with a correlation value around 1.0.

### 2.4. Permutation-based measure of CSR

Unlike ROIs selected from whole tissue sections, TMAs have many cores with regions that are folded or torn; in these cases, it appears that there are no cells present at various locations. This will result in the appearance of the intensity function of the observed point process to not be constant across the entire region, while the true underlying process may have been homogenous. Hence, use of the theoretical expected value of *K* under CSR would not be appropriate. To overcome this challenge, a permutation approach is used to obtain a robust estimate of the spatial statistic under CSR.

A class of non-parametric methods are those based on permutation or Monte-Carlo methods, where the sampling distribution of interest under the null hypothesis (i.e., CSR) is estimated by randomly assigning the labels and computing the desired statistic. This process is carried out 100 times and the resulting distribution of the statistic is an empirically derived distribution. In the context of mIF studies, each cell is labeled based on whether a certain marker is present or absent. These labels are then randomly permuted to each cell and the measure of spatial clustering is computed, maintaining the number of cells present and absent of a marker. This process is repeated a large number of times resulting in an empirically derived sampling distribution under the null hypothesis (i.e., CSR) for the computed spatial statistics (i.e., Ripley’s *K*). Using this empirical distribution, the mean is computed and used as the estimate of the spatial statistic under CSR [28] and the degree of spatial clustering is defined as the difference between the observed Ripley’s K and the mean of the empirical distribution of Ripley’s K under CSR.

### 2.5. Association of immune cell spatial clustering and survival from ovarian cancer

To measure the association of the degree of spatial clustering of immune cells with clinical outcome, while also accounting for the abundance level, Cox proportional hazards models were used. Accounting for the abundance level is critical in assessment of the spatial component as accurate estimation of the spatial measure requires an adequate number of positive cells (i.e., negative relationship between abundance level and spatial clustering value, **S1 Fig**). That is, it is not possible to estimate spatial measures when there is no positive cells and the estimate of the level of spatial clustering is somewhat related to the number of positive cells. In computing Ripley’s *K*, the radius *r* was set to 30. In order to provide clinically interpretable results, the continuous measurement of abundance and degree of spatial clustering were categorized (e.g., absent/low level/high level of abundance for immune marker). Typically, categorization takes place for each variable where values are assigned to groups depending on their relationship to the median, quartiles or some other threshold.

An alternate to setting the pre-defined threshold is use of the “optimal cut-point”, which is selected to maximize the test statistic of interest [29,30]. From a statistical standpoint, the optimal cut-point is “data-snooping” since we are allowing the results from the statistical test to inform creation of categories of the variable [31]. On the other hand, for discovery and hypothesis generation purposes, it is clinically useful to determine an optimal cut-point that can be validated in other studies with other technologies. For this study, we have chosen to use the optimal cut-point approach based on 10-fold cross-validation to determine thresholds that produce the largest difference in the survival curves. The median of the optimal cuts for abundance and degree of clustering were then used to determine the cut-points for abundance and spatial clustering for the final set of analyses (i.e., 5 groups: no immune cells; low abundance and low spatial clustering; low abundance and high spatial clustering; high abundance and low spatial clustering; and high abundance and high spatial clustering). A challenge in determining the optimal cut-points is that the number of samples in a group/category can get very small when categorizing across multiple variables. Thus, in the analysis to determine the association of immune cell abundance and spatial clustering, optimal cut-points were determined based on a grid search where the search was constrained to possible cut-points in which each group had at least 10 samples (after removing samples with zero abundance). This constrained approach ensured that each group had an adequate size for model stability.

For the co-occurrence analysis involving two types of immune cells with OS, the bivariate version of Ripley’s *K* was used. As bivariate Ripley’s *K* is only estimable when both types of immune cells are present in a sample, the level of spatial clustering was only computed when both immune markers were present. Hence, five categories were defined as AAN, APN, PAN, PPL, and PPH, where the first letter corresponds to the (A)bsence or (P)resence of the first cell type, the second letter represents the (A)bsence or (P)resence of the second cell type, and the last letter describes the level of spatial clustering (N)one/(L)ow/(H)igh. The marker was defined as present if at least one cell was positive for the immune marker. Note that the bivariate version of Ripley’s *K* is not a symmetric statistic. That is, analysis is first completed using the cytotoxic T-cell as the “anchor” or center for the computation of Ripley’s *K*, followed by the analysis using the regulatory T-cells as the “anchor”.

Using these pre-defined categories for the abundance and degree of spatial clustering, association of each cell type and phenotype with overall survival (OS) following EOC was completed using Cox proportional hazard models for CD3+ (T-cells), CD3+CD8+ (cytotoxic T-cells), and CD3+FOXP3+ (regulatory T-cells). The model included clinical covariates of age at diagnosis and stage (I, II, III, IV) and accounted for the repeated measurements per tumor/subject. Analysis was completed separately for the intratumoral ROIs and TMAs. Analyses performed for TMAs were based only on the immune marker data in the tumoral compartment of each core in order to compare findings between the intratumoral ROIs and the TMAs (i.e., restricted analysis to the cells determined to be tumor based on PanCK marker and removed the cells within the stromal compartment). Likelihood ratio tests were used to compare survival models with and without the spatial information. Permutations were performed with the package *spatialTIME* [32], and all statistical analyses were completed using RStudio with R v4.1.1.

## 3. Results

### 3.1. Quality control and assessment

Prior to statistical analysis of the mIF data, quality assessment of the data was completed. In calling a cell as positive or negative for a marker, a machine learning algorithm within the HALO (Indica Labs, New Mexico) software was used where a threshold was determined based on the intensity measurements. As the sensitivity and specificity of this method is not 100%, there may be cases where a cell is classified for multiple phenotype combinations that are not possible, such as a cell being classified as both a cytotoxic T-cell and a regulatory T-cell (**S2 Fig**). For this analysis, between 1 and 87 cells on 134 of the 260 ROI samples, and between 1 and 73 cells on 67 of the 263 TMA cores were identified as being both cytotoxic T-cell and Treg. The locations of these cells were retained but phenotype identification was considered to be CD3+ only.

### 3.2. Estimate of degree of spatial clustering and CSR in TMAs and ROIs

**Fig 2:**
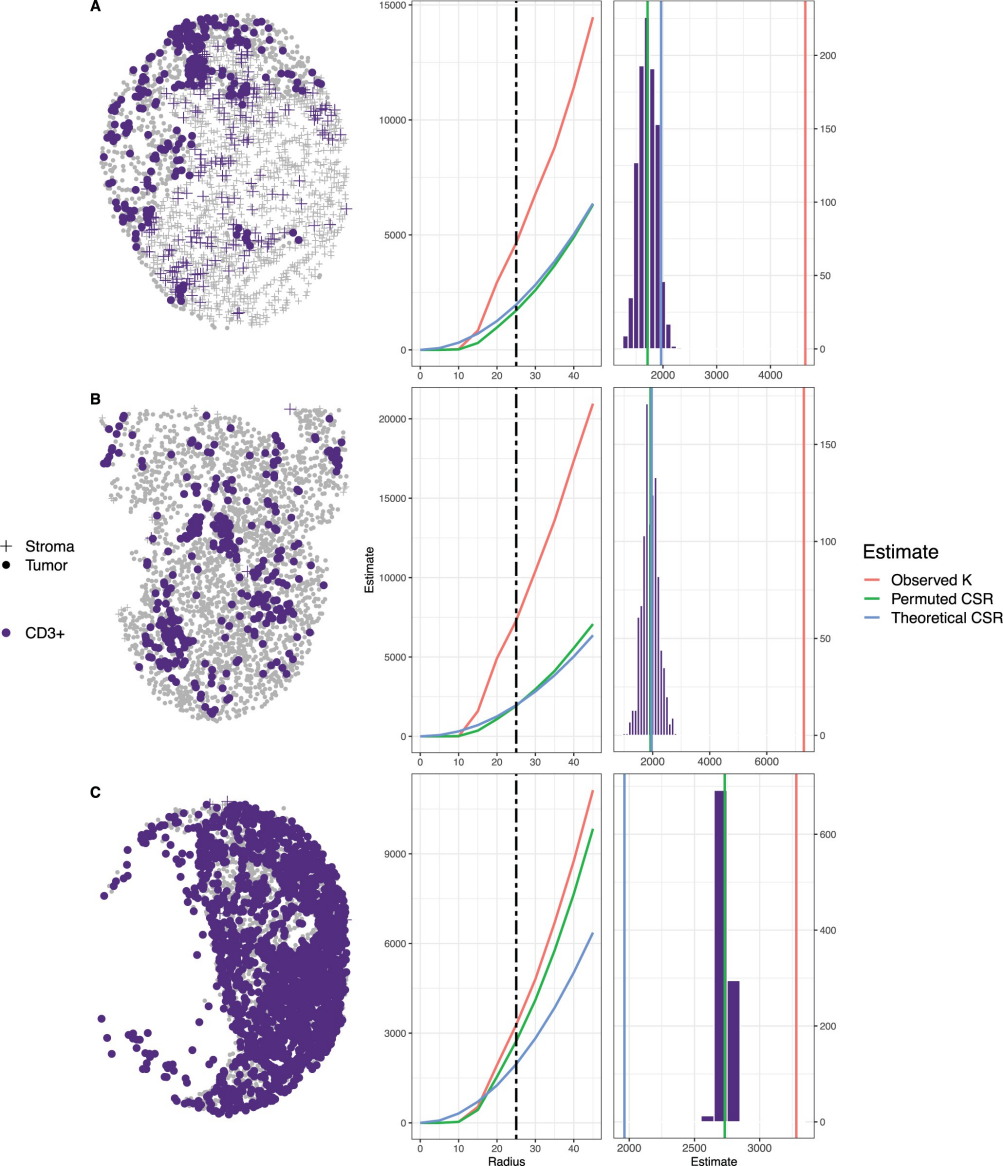
The empirical distribution of Ripley’s K under CSR generated by permutation with the translational edge correction for r = 50 and 100 for CD3+ cells. The observed value of Ripley’s K is represented by blue dotted line, the mean of the permutation-based CSR distribution is represented by the vertical black solid line, and the theoretical CSR computed assuming no “holes” in the tissue sample image is the vertical red line. As the level of missing cells increases (i.e., more “holes”) there is larger difference between the estimate of CSR based on theoretical computation using area and the estimate of CSR based on the mean of the empirically derived distribution.

Following quality control, the levels of spatial clustering were estimated using the permutation-based value for CSR, where the degree of spatial clustering was computed as the difference in the observed Ripley’s *K* and the mean of the empirical distribution of Ripley’s *K* under CSR. **Fig 2** shows three TMA cores and the empirical distribution of Ripley’s *K* under CSR for marker CD3. The first row (A) corresponds to a TMA core that does not have large areas where cells are absent, the second row (B) displays a TMA core with a moderate number of missing cells, and the third row (C) shows a TMA core with an extensive level of missing cells. As the level of missing cells increases, the difference between the theoretical and permutation-based estimates for Ripley’s *K* under CSR increases. Similarly, the histogram of the distance between the permutation-based estimate of CSR and theoretical estimates of CSR is presented in **S3 Fig** for the ROIs and TMAs. As expected for the ROIs there is not a systematic bias as there is a low level of “missing cell” regions. However, in the case of TMAs there was a systematic bias in which the mean of the difference (Theoretical estimate of CSR–Permuted estimate of CSR) is negative, indicating underestimation of Ripley’s K under CSR based on the theoretical estimate. This bias would result in a biased estimate of the degree of spatial clustering and incorrect association results with the phenotype of interest. In contrast, the empirical estimate of Ripley’s *K* under CSR would provide a more accurate measure of spatial clustering which accounts for the unevenness in the cell distribution measured in the sample. Lastly, the permutation approach allows for the assessment of spatial clustering within the tumor and/or stroma compartments separately.

### 3.3. Analysis of spatial clustering using univariate Ripley’s *K* and ovarian cancer survival

**Fig 3:**
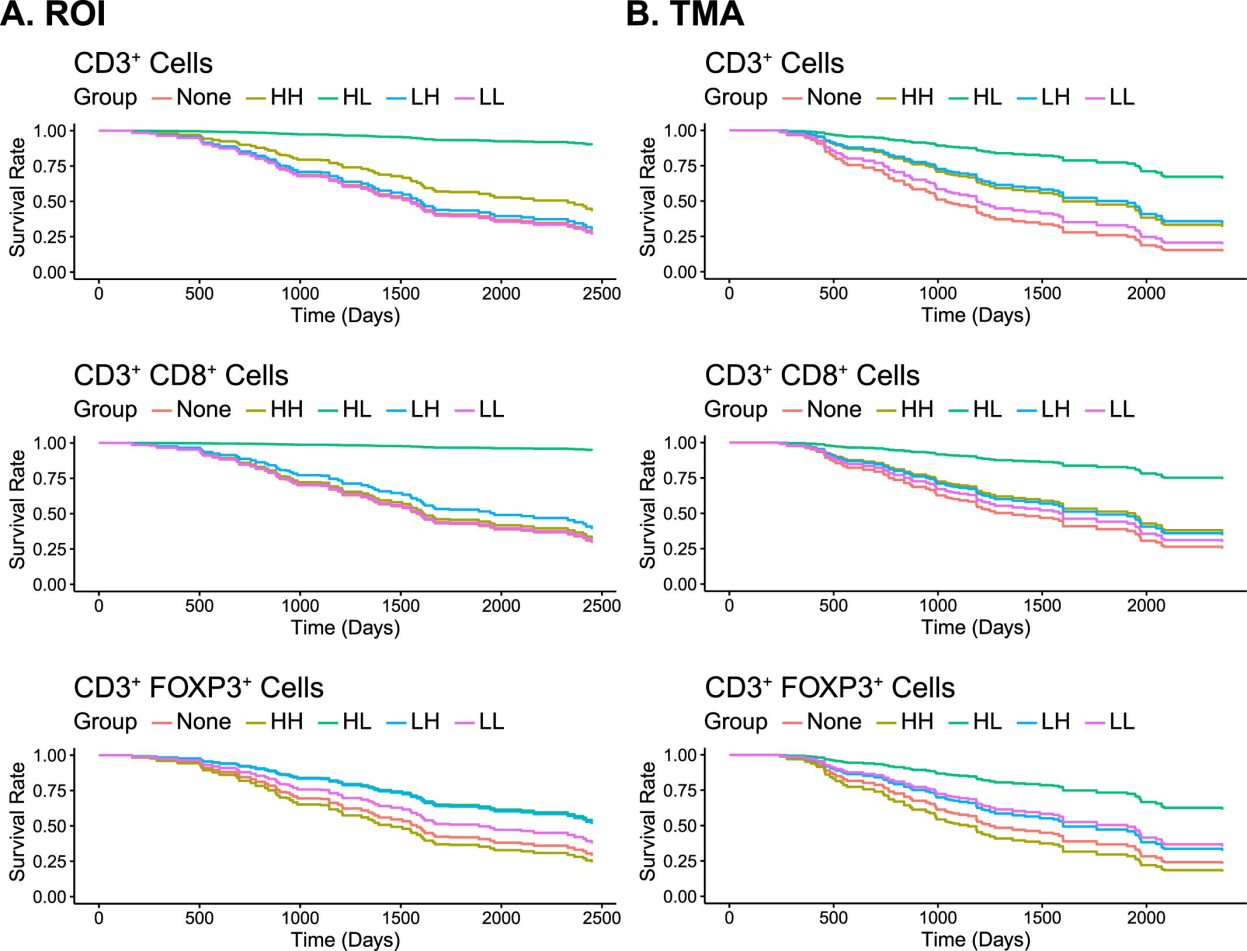
Predicted survival curves from Cox proportional hazard models for the CD3+, CD3+CD8+, and CD3+FOXP3+ cells where the degree of spatial clustering was based the permutation-based estimate of Ripley’s K under CSR (i.e., observed Ripley’s K–the mean of the empirical distribution of Ripley’s K under CSR); **(A)** results from intra-tumoral ROIs (93 subjects, 260 samples); **(B)** results from tumor compartment of TMAs (94 subjects, 263 samples). Models adjusted for age at diagnosis and stage within a repeated measures analysis framework.

Cox proportional hazard models were fit to assess the association of the abundance and spatial clustering of cells positive for CD3+ (T-cells), CD3+CD8+ (cytotoxic T-cell), and CD3+FOXP3+ (regulatory T-cells), adjusting for age at diagnosis and stage, where the degree of spatial clustering was measured by the difference of the observed estimate of Ripley’s *K* from the permutation-based estimate of Ripley’s *K* under CSR. The estimates of abundance and univariate spatial clustering were collapsed into five categorial groups as described in **Section 2.5**. **Table 1** presents the hazard ratio (HR) estimates for the different groups with the “None” group representing the reference group for the ROIs and the TMAs. **Table 1** also present the p-values (4 df) for the association of the level of spatial clustering and immune cell abundance collectively with survival. Predicted survival curves for the five groups and the three cell types are presented in **Fig 3A** for ROIs and **Fig 3B** for TMAs**. S4 Fig** presents the corresponding cumulative event curves, highlighting the survival benefit observed in patients with high abundance and low spatial clustering of T cell markers. The optimal cut-point for determining high and low abundance (based on 10-fold cross-validation) was around 1–4% for the various cell types. Using these optimal cut-points for abundance (high vs low) and degree of spatial clustering (high vs low), there was significant evidence that EOC patients with high abundance and low spatial clustering (HL group) of CD3+, CD3+CD8+, and CD3+FOXP3+ cells in their tumors had the best OS in both the ROIs and the TMAs (i.e., the “HL” group had the smallest hazard ratios (HRs) and these HRs were statistically different from the “none” group (i.e., reference group)).

When restricting the analysis to those samples with high abundance based on the optimal cut-point, a significant difference was observed in the survival curves between patients with low and high spatial clustering of CD3+ (ROIs: HR = 8.00, 95% CI (1.74, 36.77); TMAs: HR = 5.18, 95% CI (1.88, 14.25)), CD3+CD8+ (ROIs: HR = 19.84, 95% CI (1.80, 219.00); TMAs: HR = 4.50, 95% CI (1.59, 12.77)), and CD3+FOXP3+ (ROIs: HR = 2.61, 95% CI (1.18, 5.81); TMAs: HR = 5.27, 95% CI (1.97, 14.16)) immune cells, with EOC patients with low clustering having best survival (**Table 2, S5 Fig**). Lastly, we found the models including spatial information were significantly better than models with only the abundance level information included for CD3+ and CD3+FOXP3+ cells for both the analysis of the ROIs and TMAs (ROIs: CD3+ p = 1.1E-4, CD3+FOXP3+ p = 0.003; TMAs: CD3+ p = 3.8E-5, CD3+FOXP3+ p = 9.1E-4), while the model for CD3+CD8+ cells in ROI samples was statistically significant (ROIs CD3+CD8+ p = 7.3E-6; TMAs CD3+CD8+ p = 0.14). In summary, the results observed in the analysis of the ROIs were replicated in the TMA analysis, whereby subjects with high abundance and low spatial clustering of CD3+ and T cell subtypes (CD3+FoxP3+ and CD3+CD8+ cells) had significantly better overall survival.

**Table 1:** Results from survival analysis of the immune marker abundance and spatial clustering from the ROIs involving 93 subjects with 260 ROIs and TMAs involving 94 subjects and 263 cores. Degree of spatial clustering based on permutation-based estimate of CSR. Models were adjusted for age at diagnosis and stage. Group None is reference group. The overall p-value is the joint association of the five categorial variable based on immune abundance and spatial clustering on overall survival.

Study	Marker	Group*	HR	95% Confidence Interval for HR	p-value for difference from "None" reference group	Overall p-value (4 df)	Cut point used for group definition		Sample Size
							Abundance %	Degree of spatial clustering	
ROIs	CD3	HH	0.599	[0.34,1.04]	0.070	**1.0E-07**	2.9	3016	81
		**HL**	**0.067**	**[0.01,0.34]**	**0.001**				23
		LH	0.915	[0.56,1.5]	0.724				103
		LL	1.039	[0.62,1.74]	0.885				23
		None	1.000	reference	1.000				30
	CD3+ CD8+	HH	0.909	[0.49,1.7]	0.765	**5.0E-08**	3.7	4885	20
		**HL**	**0.034**	**[0,0.3]**	**0.002**				18
		LH	0.715	[0.44,1.15]	0.167				103
		LL	0.973	[0.58,1.62]	0.917				48
		None	1.000	reference	1.000				71
	CD3+ FOXP3+	HH	1.186	[0.72,1.96]	0.504	4.1E-03	1.1	5695	21
		**HL**	**0.467**	**[0.22,0.98]**	**0.043**				32
		**LH**	**0.487**	**[0.27,0.88]**	**0.018**				50
		LL	0.751	[0.5,1.12]	0.163				74
		None	1.000	reference	1.000				83
TMAs	CD3	**HH**	**0.488**	**[0.29,0.83]**	**0.009**	**3.6E-08**	1.4	5771	87
		**HL**	**0.155**	**[0.08,0.3]**	**2.3E-08**				36
		**LH**	**0.450**	**[0.26,0.79]**	**0.005**				64
		LL	0.782	[0.49,1.25]	0.307				29
		None	1.000	reference	1.000				47
	CD3+ CD8+	HH	0.671	[0.35,1.28]	0.228	**1.7E-04**	3.1	6975	18
		**HL**	**0.176**	**[0.06,0.49]**	**0.001**				22
		LH	0.718	[0.43,1.19]	0.201				53
		LL	0.845	[0.55,1.31]	0.451				65
		None	1.000	reference	1.000				105
	CD3+ FOXP3+	HH	1.272	[0.45,3.63]	0.653	**4.9E-06**	0.9	13680	12
		**HL**	**0.275**	**[0.15,0.5]**	**2.2E-05**				38
		LH	0.716	[0.41,1.24]	0.235				24
		LL	0.645	[0.41,1.01]	0.057				68
		None	1.000	reference	1.000				121

**Table 2:** Results from survival analysis of the immune marker abundance and spatial clustering from the ROIs and TMAs determined to be high abundance. Models were adjusted for age at diagnosis and stage. Group high abundance/ low spatial clustering (“HL” group) is the reference group.

Study	Marker	Group*	HR	95% Confidence Interval for HR	p-value for difference from "HL" reference group	Sample Size
ROIs	CD3	**HH**	**8.00**	**[1.74,36.77]**	**0.008**	81
		HL	1.00	reference	1.000	23
	CD3+ CD8+	**HH**	**19.84**	**[1.80, 219.00]**	**0.015**	20
		HL	1.000	reference	1.000	18
	CD3+ FOXP3+	**HH**	**2.61**	**[1.18,5.81]**	**0.018**	21
		HL	1.00	reference	1.000	32
TMAs	CD3	**HH**	**5.18**	**[1.88,14.25]**	**0.001**	87
		HL	1.00	reference	1.000	36
	CD3+ CD8+	**HH**	**4.50**	**[1.59,12.77]**	**0.005**	18
		HL	1.00	reference	1.000	22
	CD3+ FOXP3+	**HH**	**5.27**	**[1.97,14.16]**	**0.001**	12
		HL	1.00	reference	1.000	38

* HH = high abundance / high spatial clustering; HL = high abundance / low spatial clustering

### 3.4. Analysis of co-occurrence of CD3+CD8+ and CD3+FOXP3+ and ovarian cancer survival

**Fig 4:**
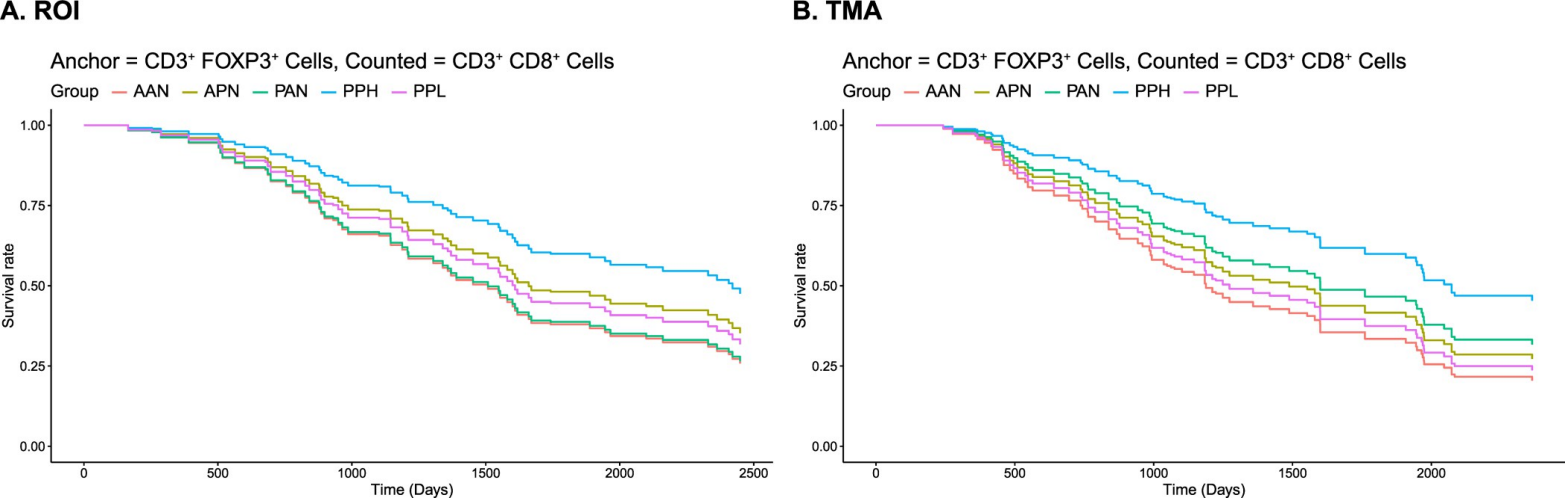
Predicted survival curves from Cox proportional hazard models for the degree of co-occurrence of CD3+FOXP3+ and CD3+CD8+ cells was based the permutation-based estimate of Ripley’s K under CSR (i.e., observed Ripley’s K–the mean of the empirical distribution of Ripley’s K under CSR); **(A)** results from intra-tumoral ROIs (93 subjects, 260 samples); **(B)** results from tumor compartment of TMAs (94 subjects, 263 samples). Models adjusted for age at diagnosis and stage within a repeated measures analysis framework.

Bivariate analysis involving Ripley’s *K* was completed to assess co-occurrence of cytotoxic T cells (CD3+CD8+) and regulatory T cells (CD3+FOXP3+) with survival following EOC, treating the CD3+FOXP3+ cell as the reference or “anchor” cell type. Results from the association of the measure of co-location with OS is presented in **Fig 4** and **Table 3**. The results using CD3+CD8+ as the reference cell were similar. Among both the ROIs and TMAs, patients with high co-occurrence of cytotoxic T-cells (CD3+CD8+) and regulatory T-cells (CD3+FOXP3+) had the best survival (ROIs: HR = 0.49, 95% CI (0.28, 0.86); TMAs: HR = 0.42, 95% CI (0.25, 0.71)). In contrast, patients with no cytotoxic or regulatory T-cells had the worst survival. Lastly, we found the model including spatial information was significantly better than a model with only the abundance level included (ROIs: p = 0.014; TMAs: p = 0.002).

**Table 3:** Results of co-localization/co-clustering of CD3+CD8+ (cytotoxic T-cells) and CD3+ FOXP3+ (regulatory T-cells) using bivariate Ripley’s K for the ROIs and TMAs. Degree of spatial clustering based on permutation-based estimate of CSR. Models were adjusted for age at diagnosis and stage. The no cytotoxic T-cells and no regulatory T-cells (none) group/category is the reference group. The overall p-value is the joint association of the five categorial variable based on immune abundance and spatial clustering on overall survival.

Study	CD3+FoxP3+ (Anchor)	CD3+CD8+	Degree of Spatial Clustering	HR	95% CI for HR	p-value for difference from the "None" reference group	Overall p-value (4 df)	Sample Size
ROI	Absent	Present	None	0.72	[0.39,1.35]	0.307	**3.2E-02**	28
	Present	Absent	None	0.98	[0.52,1.84]	0.938		16
	**Present**	**Present**	**High**	**0.49**	**[0.28,0.86]**	**0.013**		119
	Present	Present	Low	0.81	[0.45,1.47]	0.488		65
	Absent	Absent	None	1.00	reference	1.000		32
TMA	Absent	Present	None	0.77	[0.38,1.56]	0.466	**9.6E-05**	47
	Present	Absent	None	0.66	[0.33,1.31]	0.231		27
	**Present**	**Present**	**High**	**0.42**	**[0.25,0.72]**	**0.002**		96
	Present	Present	Low	0.88	[0.45,1.71]	0.702		42
	Absent	Absent	None	1.00	reference	1.000		51

## 4. Discussion and conclusion

In this research, we present a novel permutation-based analysis framework using Ripley’s *K* (univariate and bivariate) to explore the relationship between the degree of spatial clustering of immune cells with clinical outcome. The application of this framework to study of the TIME of EOC tumors from African American EOC patients revealed that not only the abundance of CD3+ and CD3+CD8+ immune cells but also the degree of spatial clustering of immune cells within the TIME were associated with overall survival. EOC patients with high abundance and low spatial clustering of tumor-infiltrating lymphocytes (TILs) and T-cell subsets had significantly better overall survival. We also observed that patients with high degree of co-localization of cytotoxic and regulatory T cells had better overall survival. Additionally, this statistical framework can be applied to mIF study involving other cancer types to understand the role of the spatial contexture of the immune cells on clinical outcome, as these findings underscore the prognostic importance of evaluating not only immune cell abundance, but also the spatial contexture of the immune cells in the TIME.

Comparison of the value of Ripley’s *K* under the assumption of CSR (complete spatial randomness) based on theoretical derivation or based on the permutation-based estimate found that the theoretical value can be a biased estimate the true value of Ripley’s *K* under CSR, with the bias more pronounced as the level of missing cells or “holes” in the TMA increased. This bias would subsequently impact the downstream association analyses (i.e., incorrect hazard ratios, confidence intervals and p-values). Many of the proposed methods being used for the spatial analysis of digital pathology data, particularly in the setting of TMAs, such as nearest neighbor distances are not correcting for this “missingness” in cell measurements and thus are prone to incorrect estimation of the degree of clustering/co-localization. An additional strength of the proposed statistical framework is that the degree of clustering can be estimated for an entire TMA or ROI or focused on just the tumor or stromal compartments.

However, there are many challenges in completing the spatial analysis of the TIME, with many areas requiring further research. One challenge in using Ripley’s *K* is selection of the proximity parameter (i.e., *r* or radius). Often, the selection of this value is based upon prior knowledge or based on practical considerations. For the present analysis of ovarian cancer tumors, we chose to use Ripley’s *K* a *r* = 30 to measure the level of clustering of immune cells in a small area (or radius). A possible choice of the proximity parameter is to compute Ripley’s *K* at several values of *r* and select the value that has the greatest difference from CSR [15,33]. However, this implementation would likely lead to different proximity parameters being used for each image and would make the spatial measure not comparable across samples. Another approach would be to treat the estimates of Ripley’s *K* as the various *r* values as a function or trajectory and applying functional data analysis (FDA), which allows for linking entire spatial trajectory to be associated with a phenotype [34–36].

Another challenge that arises when applying Ripley’s *K* is that many samples may have zero cells that express the marker of interest (i.e., “immune cold tumors”). For these cases, spatial clustering is undefined. To accommodate this case in the survival analysis, a category was defined in which samples with zero abundance and no spatial clustering was constructed. This challenge was amplified in the bivariate analysis in which both cell types had to be present in the sample for estimation of spatial co-localization. Additionally, using the optimal cut-point is a popular method for determining categories for a continuous variable (i.e., percent abundance, density), however, these methods have been shown to inflate the type I error rate [37–40] with the optimal cut-point varying between studies. Thus, we implemented a 10-fold cross-validation approach to determine the cut-point to use when categorizing the immune cell abundance and the degree of spatial clustering.

In conclusion, this paper illustrates a permutation-based approach for estimating the degree of spatial clustering when studying the TIME, with application to tumor from African American women with ovarian cancer. This approach addresses the unique challenges in the use of TMAs for studying the TIME, such as regions where cells cannot be measured due to the limitation of the sample preparation. The application of this approach also showed that in African American patients with EOC that not only the abundance but also the level of spatial clustering of T cells subtypes in the tumor is predictive of survival, where EOC patients with low level of clustering had better survival compared to patient tumors with high level of spatial clustering. We also found that co-occurrence of cytotoxic T cells with regulatory T cells conferred the best overall survival following EOC. The application of this spatial analysis framework to the study of the TIME could lead to the identification of immune content and spatial architecture that could aid in the determination of patients that are likely to respond to immunotherapies. Future research is needed to validate the findings observed in African American women with ovarian cancer in other racial/ethnic groups, along with replication of these findings in other cancer types.

## Supporting information

S1 Fig The relationship between the percent of CD3+ cells and degree of spatial clustering has an exponentially decaying relationship (A). Plots (B) and (D) (colored red in plot A), and (C) and (E) (colored green in plot A) are two ROIs which have two approximately the same percent of CD3+ but different levels of spatial clustering.
(PDF)

S2 Fig Scatter plots showing the cytoplasm intensity, which is used to classify cell positivity, for FOXP3 (Opal 540) and CD8 (Opal 570). These four plots show varying degree of phenotype misclassification and illustrates the challenge of making univariate or bivariate intensity threshold for classifying higher dimensional spaces.
(PDF)

S3 Fig Histogram of the difference between the theoretical estimate and the permuted estimate of CSR for the ROIs **(A)** and TMA core samples **(B)** for CD3+, CD3+CD8+ (cytotoxic T-cell), and CD3+FOXP3+ (Regulatory T-cell or Treg). The dashed black line represents zero and the solid black line represents the mean of distribution. To better visualize the distribution, the plots were scaled towards x = 0.
(PDF)

S4 Fig Cumulative event curves from Cox proportional hazard models for the CD3+, CD3+CD8+, and CD3+FOXP3+ cells where the degree of spatial clustering was based the permutation-based estimate of Ripley’s K under CSR (i.e., observed Ripley’s K–the mean of the empirical distribution of Ripley’s K under CSR); **(A)** results from intra-tumoral ROIs (93 subjects, 260 samples); **(B)** results from tumor compartment of TMAs (94 subjects, 263 samples). Models adjusted for age at diagnosis and stage within a repeated measures analysis framework.
(PDF)

S5 Fig Predicted survival curves for patients with high abundance stratified by level of spatial clustering from Cox proportional hazard models for the CD3+, CD3+CD8+, and CD3+FOXP3+ cells where the degree of spatial clustering was based the permutation-based estimate of Ripley’s K under CSR (i.e., observed Ripley’s K–the mean of the empirical distribution of Ripley’s K under CSR); **(A)** results from intra-tumoral ROIs **(B)** results from tumor compartment of TMAs. Models adjusted for age at diagnosis and stage within a repeated measures analysis framework.
(PDF)

S1 Table Summary of the demographics of the African American women with EOC in AACES included in the study.
(XLSX)
