# Supplementary figures and images for: Tumor immune cell clustering and its association with survival in African American women with ovarian cancer
Source: PLoS Comput Biol. 2022 Mar 2;18(3):e1009900. doi: 10.1371/journal.pcbi.1009900 (PMC8920290; doi:10.1371/journal.pcbi.1009900)

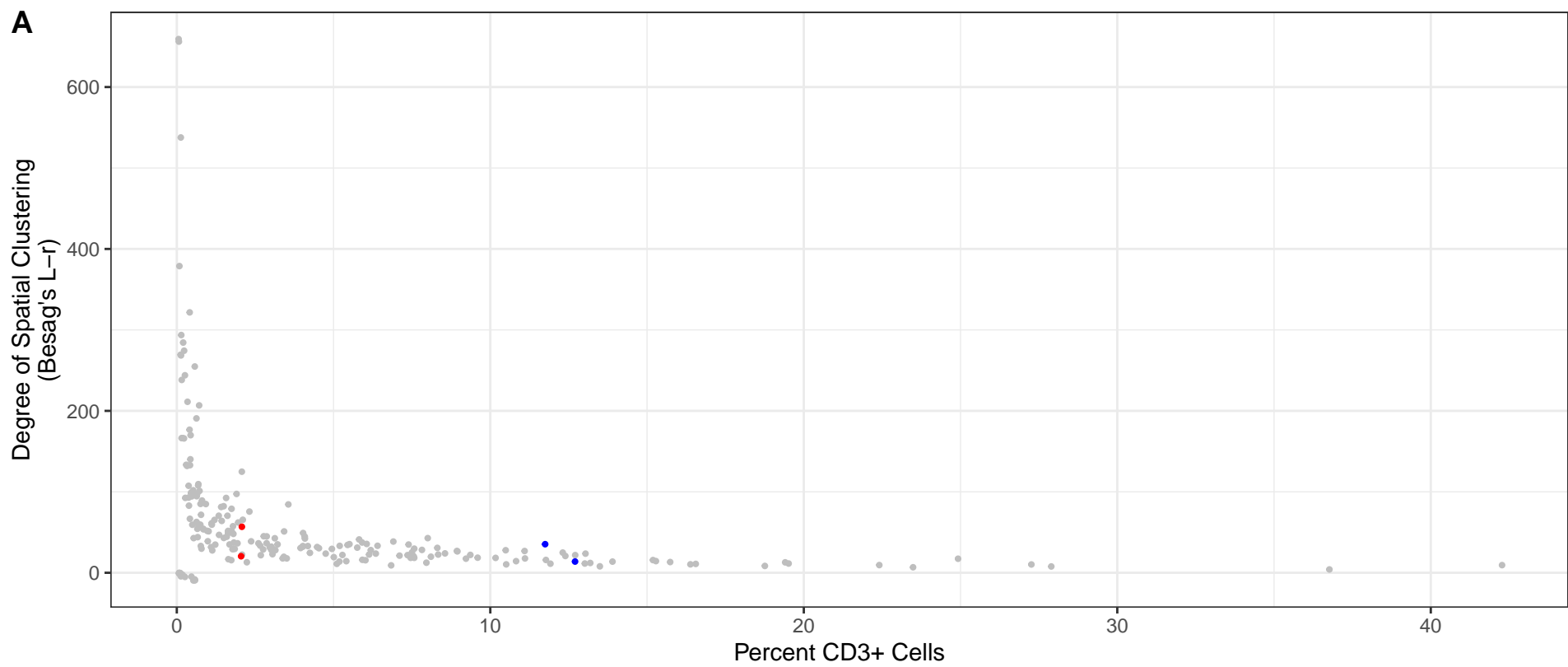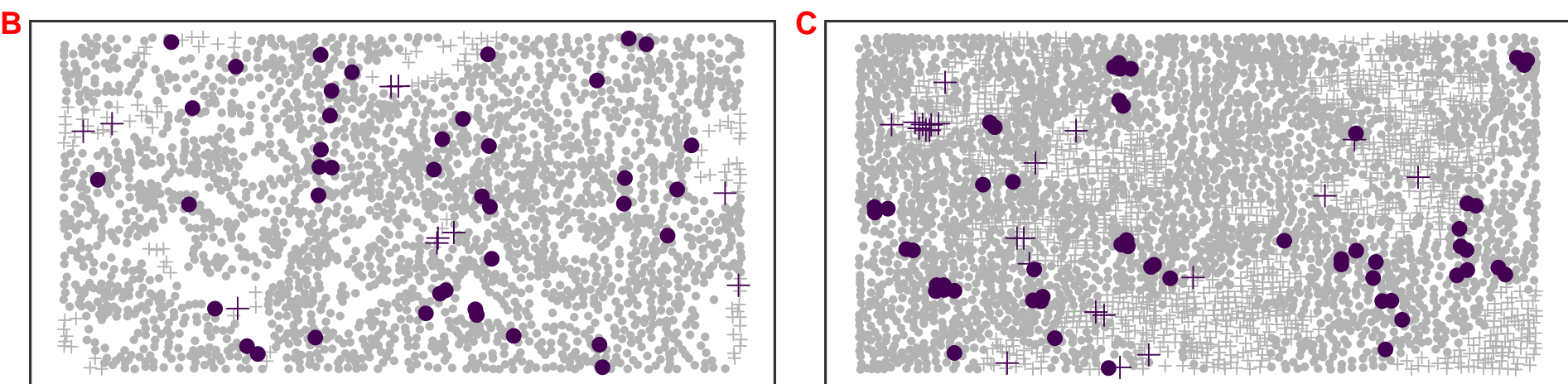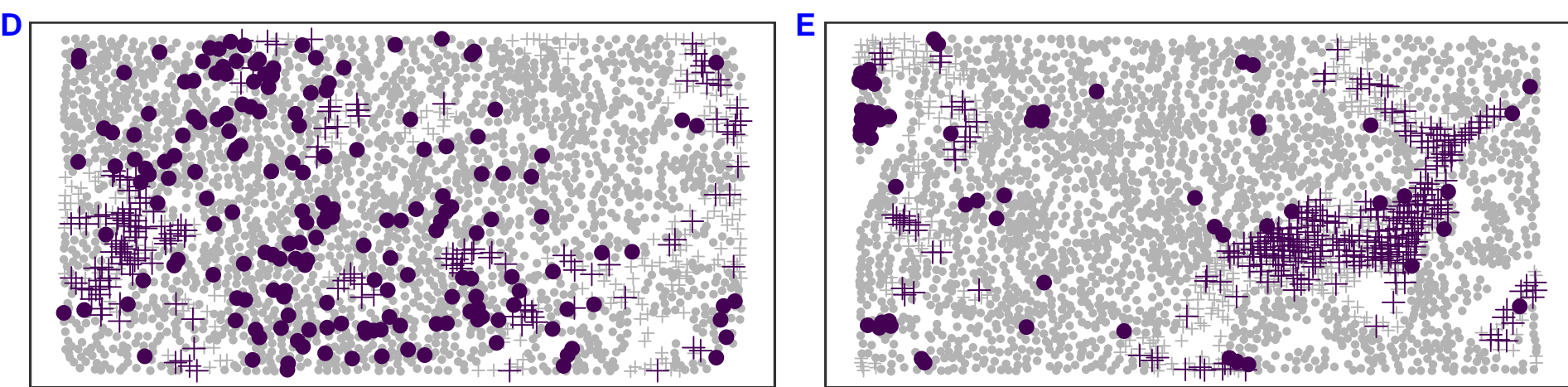

● CD3+    + Stroma    ● Tumor

Supplement: S1 Fig — The relationship between the percent of CD3+ cells and degree of spatial clustering has an exponentially decaying relationship (A). Plots (B) and (D) (colored red in plot A), and (C) and (E) (colored green in plot A) are two ROIs which have two approximately the same percent of CD3+ but different levels of spatial clustering. (PDF) [file pcbi.1009900.s001.pdf]

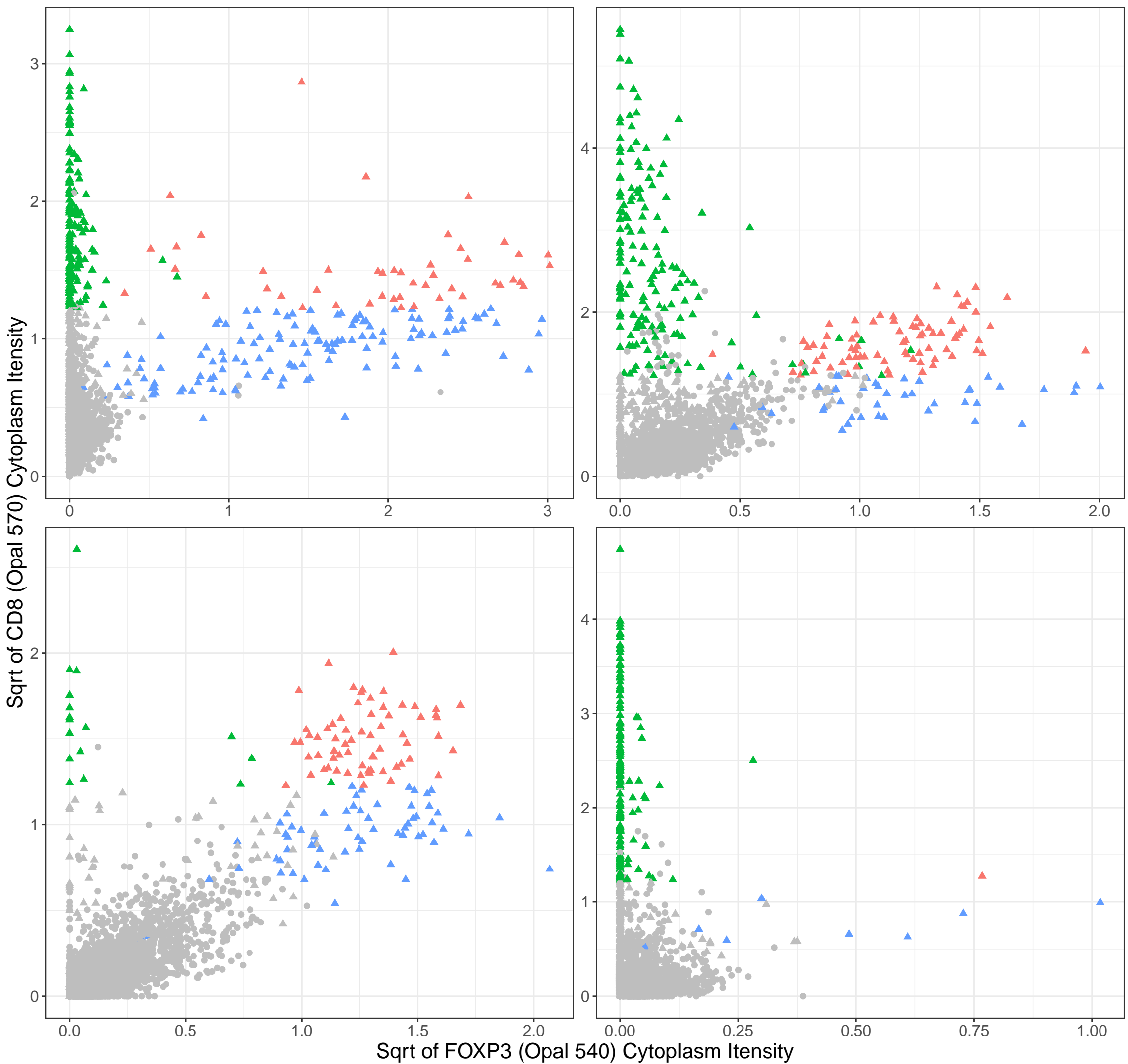

Cell Classification    ● Both CD3+ FOXP3+ and CD3+ CD8+    ● CD3+ CD8+ Only    ● CD3+ FOXP3+ Only    CD3 Classification    ● CD3-    ▲ CD3+

Supplement: S2 Fig — Scatter plots showing the cytoplasm intensity, which is used to classify cell positivity, for FOXP3 (Opal 540) and CD8 (Opal 570). These four plots show varying degree of phenotype misclassification and illustrates the challenge of making univariate or bivariate intensity threshold for classifying higher dimensional spaces. (PDF) [file pcbi.1009900.s002.pdf]

## A. ROI

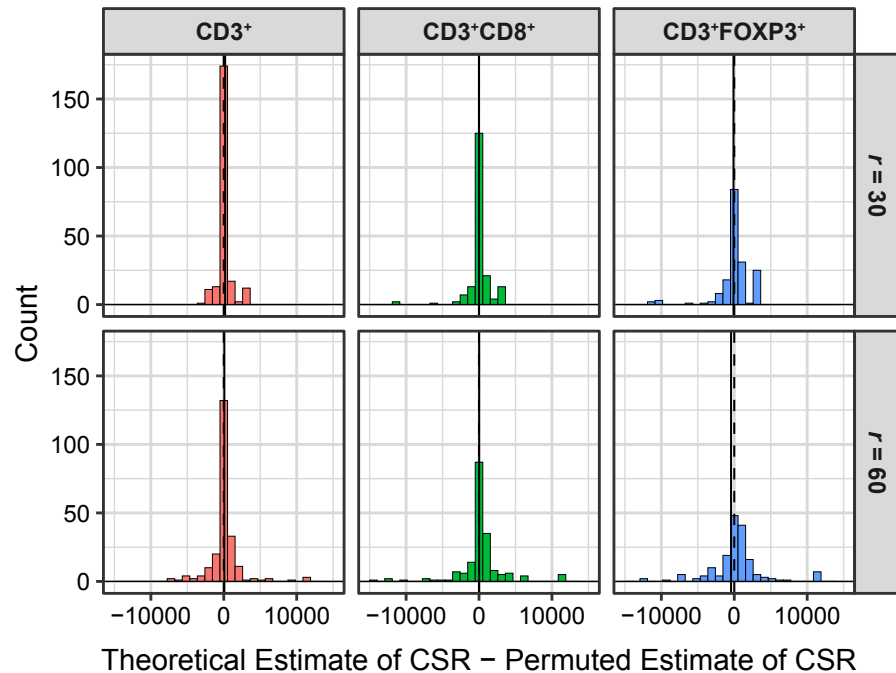

## B. TMA

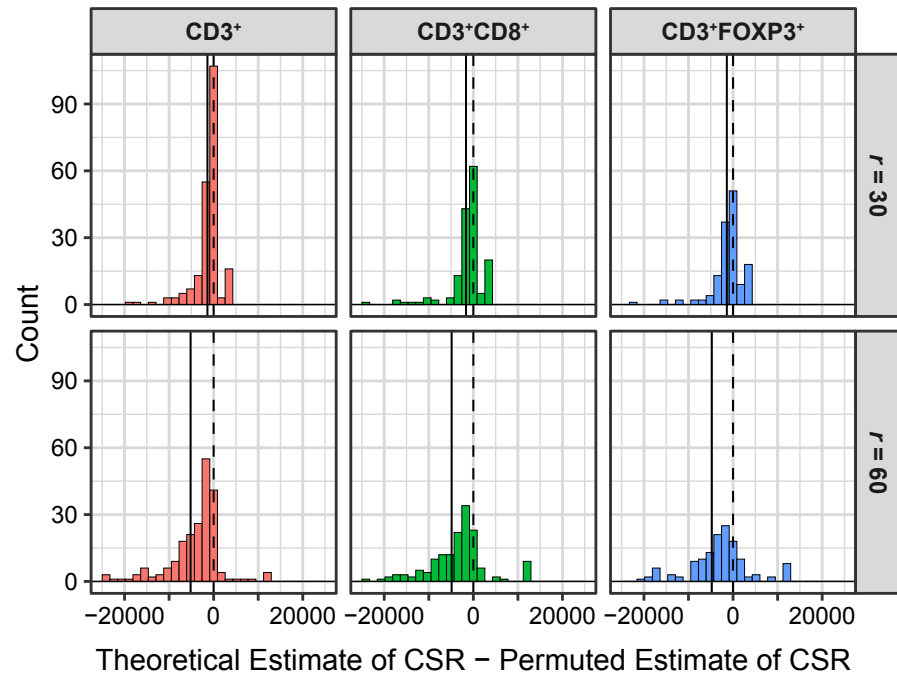

Supplement: S3 Fig — Histogram of the difference between the theoretical estimate and the permuted estimate of CSR for the ROIs (A) and TMA core samples (B) for CD3+, CD3+CD8+ (cytotoxic T-cell), and CD3+FOXP3+ (Regulatory T-cell or Treg). The dashed black line represents zero and the solid black line represents the mean of distribution. To better visualize the distribution, the plots were scaled towards x = 0. (PDF) [file pcbi.1009900.s003.pdf]
