# Supplementary material for: Tumor immune cell clustering and its association with survival in African American women with ovarian cancer
Source: PLoS Comput Biol. 2022 Mar 2;18(3):e1009900. doi: 10.1371/journal.pcbi.1009900 (PMC8920290; doi:10.1371/journal.pcbi.1009900)
Supplement: S4 Fig — Cumulative event curves from Cox proportional hazard models for the CD3+, CD3+CD8+, and CD3+FOXP3+ cells where the degree of spatial clustering was based the permutation-based estimate of Ripley’s K under CSR (i.e., observed Ripley’s K–the mean of the empirical distribution of Ripley’s K under CSR); (A) results from intra-tumoral ROIs (93 subjects, 260 samples); (B) results from tumor compartment of TMAs (94 subjects, 263 samples). Models adjusted for age at diagnosis and stage within a repeated measures analysis framework. (PDF) [file pcbi.1009900.s004.pdf]

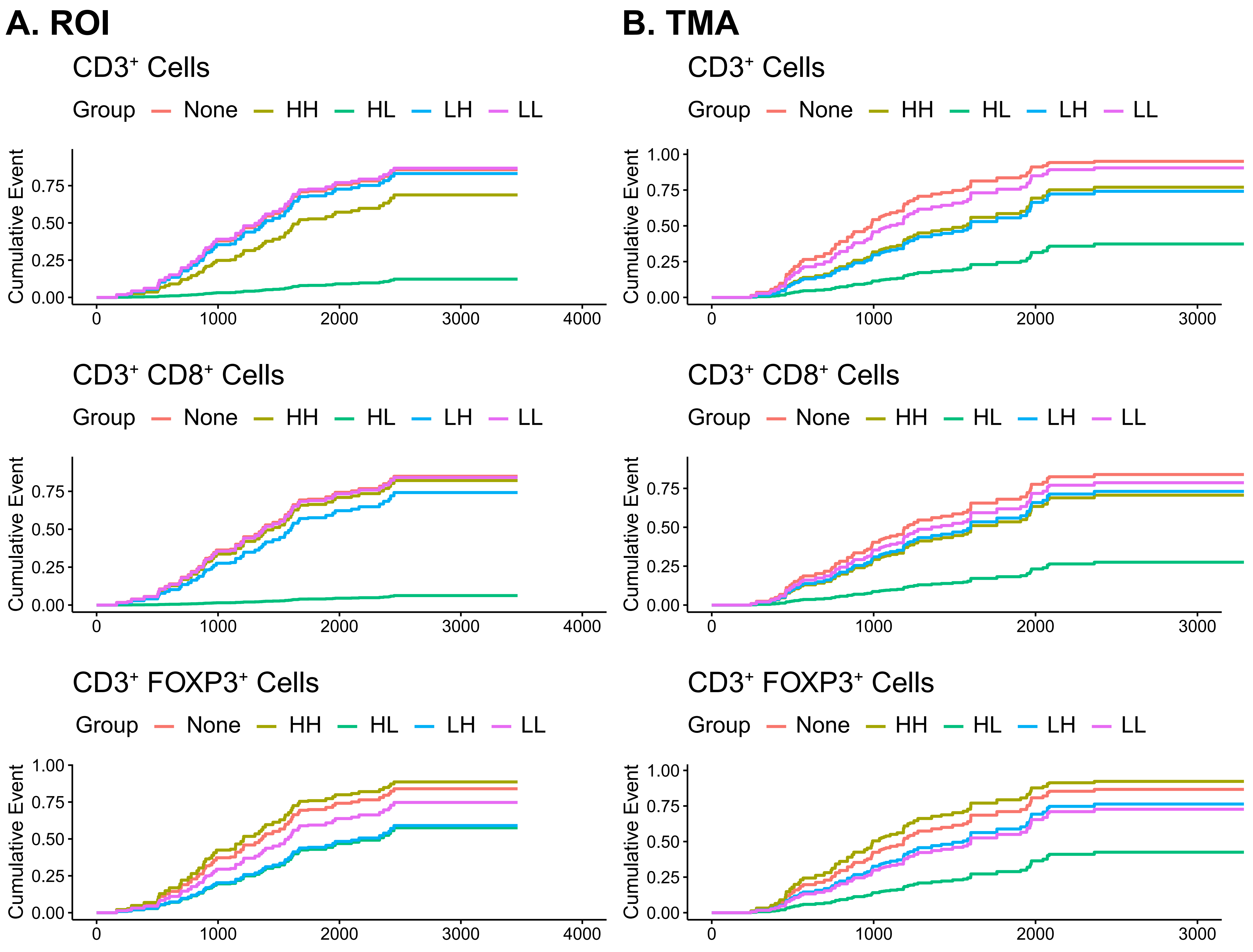
A. ROI
B. TMA
CD3⁺ Cells
Group None HH HL LH LL
Cumulative Event
0.00
0.25
0.50
0.75
1.00
0
1000
2000
3000
4000
CD3⁺ CD8⁺ Cells
CD3⁺ FOXP3⁺ Cells
