# Supplementary material for: Tumor immune cell clustering and its association with survival in African American women with ovarian cancer
Source: PLoS Comput Biol. 2022 Mar 2;18(3):e1009900. doi: 10.1371/journal.pcbi.1009900 (PMC8920290; doi:10.1371/journal.pcbi.1009900)
Supplement: S5 Fig — Predicted survival curves for patients with high abundance stratified by level of spatial clustering from Cox proportional hazard models for the CD3+, CD3+CD8+, and CD3+FOXP3+ cells where the degree of spatial clustering was based the permutation-based estimate of Ripley’s K under CSR (i.e., observed Ripley’s K–the mean of the empirical distribution of Ripley’s K under CSR); (A) results from intra-tumoral ROIs (B) results from tumor compartment of TMAs. Models adjusted for age at diagnosis and stage within a repeated measures analysis framework. (PDF) [file pcbi.1009900.s005.pdf]

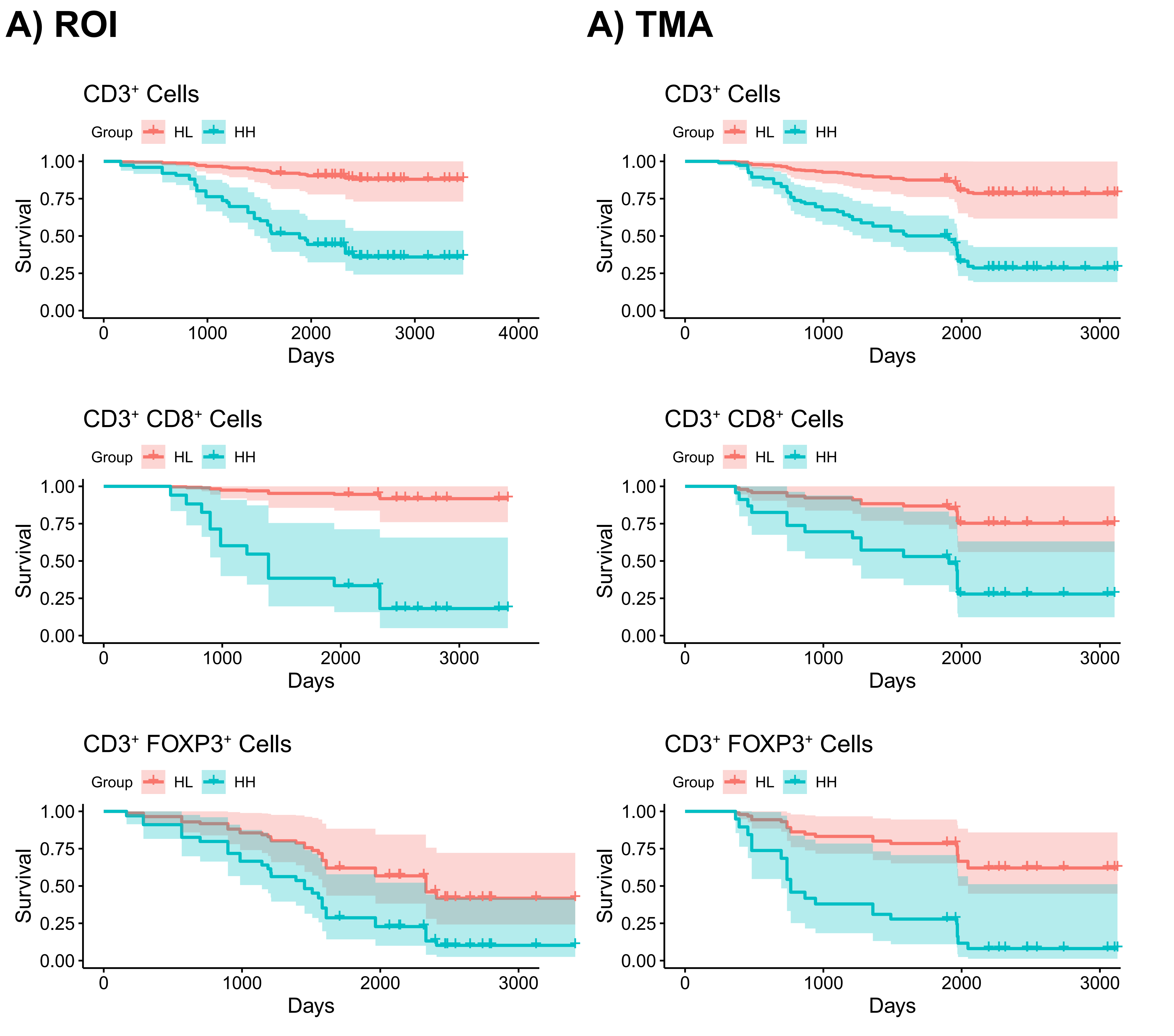
A) ROI
A) TMA
CD3⁺ Cells
Group
HL
HH
Survival
1.00
0.75
0.50
0.25
0.00
Days
0
1000
2000
3000
4000
CD3⁺ CD8⁺ Cells
CD3⁺ FOXP3⁺ Cells
